# A rare phenomenon of pregorexia in Pakistani women: need to understand the related behaviors

**DOI:** 10.1186/s40337-022-00589-8

**Published:** 2022-05-21

**Authors:** Tamkeen Saleem, Shemaila Saleem, Sheikh Shoib, Jaffer Shah, Syeda Ayat-e-Zainab Ali

**Affiliations:** 1 Department of Psychology, International Islamic University, Islamabad, 44000 Pakistan; 2 Department of Physiology, Federal Medical and Dental College, Islamabad, 44000 Pakistan; 3 Department of Psychiatry, Jawaharlal Nehru Memorial Hospital (JLNMH), Rainawari, Srinagar, Jammu and Kashmir 190003 India; 4 Medical Research Center, Kateb University, Kabul, Afghanistan; 5 Department of Health Services, New York State Department of Health, New York, USA

**Keywords:** Disordered eating, Eating disorders, Pregnancy, Pregorexia

## Abstract

**Background:**

In Pakistan, for a decade or so, there has been a huge increase in body ideals, and thinness and eating disorders reported during pregnancy. The purpose of the present research was to study the lived experiences and behaviors characterized by pregorexia in Pakistani young women.

**Method:**

A phenomenological approach was used to study eating disorder-related behaviors among pregnant women. A criterion sample of 15 women (22–34 years of age) having difficulty with their body image, the decline in caloric intake, skipping meals, and extensive exercise from a private gynae clinic of Islamabad was selected. The participants were screened on the DSM-5 criteria of anorexia nervosa. They were also asked questions about their diet intake, behaviors executed to lose or maintain weight, use of any medical or chemical substance to control weight, any social activities/behaviors, type of exercise if any, duration or frequency of exercise, and behaviors that made them feel better.

**Results:**

The findings revealed that 93.33% of women met the complete criteria of Anorexia nervosa. 86.6% had never been diagnosed or treated with anorexia nervosa, however, 13.33% were diagnosed with anorexia nervosa comorbid with depression. Results indicated a likelihood of having anorexic tendencies in 40% of women and the other 60% developed the symptoms during pregnancy only. Behaviors related to pregorexia were found to be: skipping meals, eating small portions, control on calorie intake, following diet plans available on YouTube, taking fat burn tea, avoiding the presence of elders of the family while taking meals, eating alone to limit food portion, taking laxatives or medicine to control weight, induced vomiting after eating, eating to match the norm of eating (supervised eating by elders) being pregnant and later self-induced vomiting, or eating slowly and consuming more time, pretending to eat the suitable amount of food. Women also engaged in fast walking, light exercise, and intensive cardio to control weight and stay in body shape. Around 86% reported that controlling weight, calorie intake, and exercising made them feel better.

**Conclusion:**

There is a need to understand and differentiate pregorexia from other eating behaviors and problems among pregnant women with respect to cultural context.

## Plain English summary

Pregorexia is a term used to describe a condition where an individual displays a heightened fear of pregnancy-related weight gain. The present study is the first to examine this eating disorder in Pakistan. Some behaviors related to pregorexia were commonly identified to include skipping meals, eating small portions, following diet plans available on YouTube, eating alone to limit food portion, taking laxatives or medicine to control weight, induced vomiting after eating, and pretending to eat the suitable amount of food. We recommend clinicians interacting with pregnant women to screen for these behaviors when taking histories to provide better support, education and increased awareness.

## Introduction

Adequate nutrition and a healthy diet are necessary at every stage of life and even more important during pregnancy as there is a need to ensure the optimal birth of a baby, fetus development, and maternal health [35]. A pregnant woman is most certainly faced with her biological femininity, which influences her self-concept. In addition, she experiences a number of specific apprehensions concerning the course and outcome of pregnancy. This makes her particularly vulnerable as she is conditioned by the adaptive capacities of her personality, lifestyle, and status in the social environment [5]. During pregnancy, the body of the woman is subject to vigorous alterations which are related to the needs of baby development. However, these alterations may bring physical and psychological distress for the pregnant woman [6, 11, 16].

Eating disorders are a major public health concern for adolescent girls and young women [27]. It may be said that feelings of not having control over the body during pregnancy may trigger underlying tendencies of disordered eating. It is also possible that such tendencies do exist even before conception [29].

Weight gain during pregnancy may be alleged as an enhancement in femininity or just the opposite because the woman may feel a compromise on her attractiveness. In the modern-day people are so much exposed to media and it has become a significant tool to shape the perceptions and behaviors of people. The desire to lose weight is strongly connected with poor body image which is developed over a lifetime from influences of various agents like family, peers, media, and social pressure. Media has an overall negative effect on an individual’s body image and esteem [19]. Media portrays various rigorous messages promoting slim bodies, which are often away from the reach of the bulk of women. Thus, as a consequence, they feel body dissatisfaction. The same may occur during pregnancy and women may experience abnormal health behavior or eating disorders, mainly anorexia nervosa in pregnancy [14].

Pregnancy anorexia nervosa is a new psychology notion that was described as “Pregorexia” first time in 2008 by the media. It is defined as an eating disorder of pregnant women who tend to restrict their dietary caloric intake and perform intensive physical exercise and activities [21, 36]. Pregorexia is anorexia nervosa that comes about during the period of pregnancy; pregnancy may be an activating element for this eating disorder [24]. The symptoms and behaviors for pregorexia are typical of anorexia nervosa. Women with pregorexia repetitively examine their weight and take various harmful actions to reduce it for instance: restricting diet or starvation, use of laxatives and medicines, intensive physical exercise, and purging [18, 26]. A study regarding pregorexia indicated that women feel pressured to stay slim during pregnancy and postpartum [34].

Pregorexia is not formally recognized by American Psychological Association as a mental disorder but the term may be considered as an eating disorder during pregnancy. Because the symptoms cause threatening problems for both mother and baby like abortion, low birth weight, pre-term birth, microcephaly, maternal hypertension, and anemia [38]. There is much more need for qualitative and quantitative research to bring awareness about eating disorders, specifically pregorexia.

In Pakistan, there has been a shift from the traditional eating patterns and ideals of thinness due to advent of international fast-food chains (McDonald's, KFC, Subway, Hardees, One Potato Two Potato, Pizza Hut, Dominoes, etc.). mushroom growth of health businesses (gyms and slimming clinics}, lifestyle change gurus, and cosmetic surgery clinics (stomach, facial, stomach patching, intestinal reduction), print and electronic media portrayal of thin female models [19]. There is no such study connecting body image and pregnancy in Pakistan. However, it may be speculated that these factors influenced the overall women in the country, making the pregnant women vulnerable to maintain their figure and ideals of beauty, thinness and body image. Researchers have reported that Pakistani women are similar to British women (White and Asian) in aspiring for a thinner "Western Ideal" body [4]. A study reported that pregnant women have dissatisfied self-esteem [40]. A study reported that both social and psychological factors contribute to body image changes and body dissatisfaction during pregnancy [33]. Keeping the lifestyle changes and the pressure for "Thin and Fit", it is speculated that such trends may be prevalent among women during pregnancy which requires a probe in Pakistani pregnant women.

Further, the research on nutritional data and practices among pregnant women in developing countries does provide some empirical evidence. However, such information in Pakistan is limited [23] and almost a nullity for eating disorders during pregnancy. Therefore, the present research aimed to study the lived experiences and the behaviors characterized by pregorexia in Pakistani young women who reported having symptoms similar to anorexia nervosa during pregnancy.

## Method

A phenomenological approach was used to study the related behaviors among pregnant women. For this purpose, a criterion sample of 15 women who reported in a private gynae clinic of Islamabad regarding having difficulty with their body image, the decline in caloric intake, skipping meals, and extensive exercise was selected to study the lived experiences related to anorexia nervosa during pregnancy. The women participants who reported at the clinics were underweight before their pregnancy and had not even gained much weight of 2 kg during their first trimester which was supposed to be increased as per the criteria of American Institute of Medicine. Considering their complaints, women were assessed and identified by clinicians to be having pregorexia. The 15 women with ages range between 22 and 34 years were interviewed to study the behaviors related to pregorexia. All women had a single fetus pregnancy. The participants were screened on the DSM 5 criteria of anorexia nervosa (Table 1).

**Table 1:** Demographic and symptom characteristics of participants (N = 15)

	*N* (%) or mean ± SD
Age	26.93** ± **3.82
Working women	3 (20%)
*Education*	
BS/BA/BSc	11 (26.6%)
Masters	4 (73%)
*Years of marriage*	
1	10 (66.66%)
1–2	3 (20%)
3>	2 (13.33%)
First pregnancy	8 (53.33%)
*Trimester*	
1st	9 (60%)
2nd	6 (40%)
No. of interviews	2.33 ± 0.48
*History of abortion*	
Yes	6 (40%)
No	9 (60%)
*DSM-5 criteria for anorexia nervosa*	
A. Restriction of energy intake relative to requirements, leading to a significantly low body weight in the context of age, sex, developmental trajectory, and physical health. Significantly low weight is defined as a weight that is less than minimally normal or, for children and adolescents, less than minimally expected	15 (100%)
B. Intense fear of gaining weight or of becoming fat, or persistent behaviour that interferes with weight gain, even though at a significantly low weight	15 (100%)
C. Disturbance in the way in which one’s body weight or shape is experienced, undue influence of body weight or shape on self-evaluation, or persistent lack of recognition of the seriousness of the current low body weight	14 (93.33%)
Total participants meeting full criteria for anorexia nervosa	14 (93.33%)
*Specification of type*	
Restricting type: During the last three months, the individual has not engaged in recurrent episodes of binge eating or purging behavior (i.e. self-induced vomiting, or the misuse of laxatives, diuretics, or enemas). This subtype describes presentations in which weight loss is accomplished primarily through dieting, fasting and/or excessive exercise	8 (53.33%)
Binge-eating/purging type: During the last three months the individual has engaged in recurrent episodes of binge eating or purging behavior (i.e. self-induced vomiting, or the misuse of laxatives, diuretics, or enemas)	7 (46.66%)
*Medical problems/complaints other than anorexia nervosa*	
Anemia	9 (60%)
Dehydration	6 (40%)
Poor Nutrition	15 (100%)
Gastrointestinal Problems	5 (33.33%)
Urinary Tract Infections	4 (26.66%)
Menstruation Problems	7 (46.66%)
Anxiety	11 (73.33%)
Co-Morbid Depression	2 (13.33%)
*Anorexia nervosa symptoms occurrence/on set*	
Anorexia nervosa before pregnancy	2 (13.33%)
Anorexia nervosa during pregnancy	13 (86.66%)
Reported reluctance for pregnancy	8 (53.33)
*Exercise and physical activities*	
Duration of exercise/physical activity in Minutes	106** ± **56.78
*Specify current severity (BMI at the time of assessments)*	
Mild: BMI more than 17	4 (26.66%)
Moderate: BMI 16–16.99	10 (66.66%)
Severe: BMI 15–15.99	1 (6.66%)
Extreme: BMI less than 15	–

The participants were interviewed in detail. An interview guide was developed for the present study to assess the phenomenon of pregorexia. The interview guide was developed consulting the suitable literature, seeking the opinions of experts in psychiatry (2), psychologist (1), and gynae/obstetrics (2). The participants were asked questions about their diet intake, behaviors executed to lose or maintain weight, use of any medical or chemical substance to control weight, any social activities/behaviors, type of exercise if any, duration or frequency of exercise, and which behavior made them feel better.

It is unlikely in Pakistan that all the cases with Eating disorders reported in gynecology clinics may be referred to psychiatric clinic. People are not that much receptive. Those who tend to experience eating disorders do not visit psychiatry clinics due to stigmatization. Therefore, these women were not referred to a psychiatric clinic for evaluation by a psychiatrist. However, their diagnosis were confirmed by the trained psychologists and then the reports of history and symptoms were assessed by a psychiatrist for confirmation. In this manner, the final diagnosis of anorexia nervosa was made by the licensed psychiatrist.

For data collection, the purpose and significance of the study were communicated with the clinic authorities as well as participants in advance, and the interviews were scheduled as per their convenience. They were informed that they may leave the interview or withdraw their responses at any point in time during the study without any penalties. The face-to-face interviews were conducted in a separate quiet room without interruptions. The interviews took 30–45 min per participant. The interviews were recorded with the permission of the participants and later transcribed into notes. If the participants exhibited an emotional problem during the time of the interview, adequate psychological counseling was provided to avert any secondary psychological harm. The researchers who were involved in interviewing remained neutral in collecting data and developed a good rapport with the participants utilizing the Rogerian principles of unconditional positive regard, non-judgmental behavior, genuineness, empathy, and active listening. For each participant, 1–2 face-to-face sessions were conducted and for a few 1 telephone call was made to ensure data collection was complete and clear and also to certify data collection at multiple points.

Transcribing and data analysis were carried out for each interview within 48 h of each interview. For analysis of the data, Colaizzi's phenomenological method was used. Two researchers independently familiarized and reviewed the transcribed interviews, identified significant and meaningful statements, extracted and clustered the themes, themes integrated into the exhaustive description, structure development of the phenomenon and then finally verification of the fundamental structure by informants via discussion of the contents of the themes to validate whether the themes captured their original experiences. Data saturation level was verified by two independent researcher in a process carried out in parallel with data collection. Data saturation was assessed by data content coverage. However, to bring objectivity, data saturation level was verified by two independent researchers in a process carried out in parallel with data collection—then, saturation was based on consensus between both researchers that no new information and themes were available.

## Results

The table reports that present study was conducted with a sample of 15 pregnant women having their first pregnancy with the age range of 22–34 years (Mean Age = 26.93). There were 20% working women, 11% had completed their BS and 4% had done their masters, 66.66% had less than 1 year of marriage duration, 20% had experienced 1–2 years of marriage and only 13.33% had 3 and more years of marriage. All women reported it to be their first pregnancy, 60% were having their First trimester and 40% were having their 2nd Trimester. Among all women, 40% reported a history of abortion (Table 1).

Regarding the symptoms 93.33% met the full criteria of anorexia nervosa, 53.33% had the restricting type of anorexia nervosa and 46.55% had the Binge-eating/purging type. It was reported by 13.33% of women that they had symptoms prior to pregnancy and they had depression for which they were seeking help, however, 86.66% of women reported that they developed anorexic symptoms during pregnancy as they could not control their ever-increasing weight. Some women also reported a reluctance for pregnancy i.e. 53.33%

Symptoms other than anorexia nervosa were also studied and results showed that other symptoms or medical problems included Anemia (60%), Dehydration (40%), Poor Nutrition (100%), Gastrointestinal Problems (33.33), Urinary Tract Infections (26.66%), and Menstruation Problems (46.66%). Psychological problems reported by women included anxiety (73.33%) and Depression (13.33%).

The pregnant women reported the following behaviors related to pregorexia; skipping meals, eating small portions, control on calorie intake, following diet plans available on youtube, taking fat burn tea, avoiding the presence of elders of the family while taking meals, avoiding eating in gatherings, eating alone to limit food portion, taking medicine to control weight, induced vomiting after eating, eating to match the norm of eating (supervised eating by elders) being pregnant and later self-induced vomiting, or eating slowly and consuming more time, pretending to eat the suitable amount of food (Table 2). Women also indicated physical activities and exercise to control weight and stay in body shape which included fast walking, light exercise, and intensive cardio. Most of the women reported that taking meals by controlling calorie intake and exercising made them feel better.

**Table 2:** Themes Related to Behaviors connected to Pregorexia identified through interviews

Themes	Sub-themes	Quotations
Meal avoidance	Skipping meals	“I avoid taking my meals” “I over sleep to avoid taking meals” “I keep myself busy, so that my focus does not go to food items” “I starve myself and do not eat.”
	Eating small portions	“I take 8–9 snacks, as youtube videos show that small portion meals do not make one put on weight”
Diet control	Control on calorie intake	“I count my calories for food that I learned in weight training program” “The guide to stay lean is to keep an eye on calories you take in” “I eat less not only because I feel nausea but also to curb my upsetting thoughts related to weight gain”
	Following diet plans	“I follow diet plans available on youtube I eat healthy” “I make diet plan in my mind and count calories”
Use of products to control weight	Taking medicines or herbal remedies to control weight	“Although I know medications are not good for baby in the womb but I can’t help it. I feel pressured to take medicines for the weight control. I have tried herbal pills (Garcinia Cambogia) and pills recommended (Di-Urate, Ezcol etc.) in online videos and websites. My family doesn’t know about it” “Its easy to order the medicines, tonics and teas online, along with that I take some vitamins as well that keep me healthy. It is self-prescribed not consulted with my doctor” “I take medicines for weight control. I have tried different medicines at different timies (bisacodyl, green coffee bean capsules, orslim)”
	Taking fat burn tea	“I take green tea” “I follow some nutritionists on youtube and follow their fat cutting tea and roti. It really helps. I have been following that from last two years”
Induced vomiting after eating	Vomiting as a tool to control weight	“I often feel choking after eating and then I puke” “I try to throw up any additional quantity with the fear of deshaping my body. I put my finger in my throat and it makes it easy” “When I am distressed by thoughts of being Fat I purge and vomit out” “Sometimes I eat and eat but then I puke everything out and then again after sometime I find myself in eating something” “There is already terrible nauseated feelings and episodes of vomiting that self-induced vomiting is not an issue”
Physical activities and Exercise	Physical activities and exercise to control weight and stay in body shape	“I do fast walking, light exercise and at time intensive cardio as I don’t want to lose my body shape” “I understand that exercise can be harmful for fetus but I feel active after it and my body is important so I do light exercise, walk and household tasks like mopping floor (standing)” “Exercise makes me feel good. It helps in controlling my worry of getting fat” “I take light exercise, stretching and go for swimming for a few minutes. I walk for 2–3 h”
Body dissatisfaction	Worry to lose shape	“I am worried about my body shape and if I would be able to get my body back or not” “I have severe perinatal anxiety, I fear for the loss of baby as well as for stretchmarks and disfiguring” “I feel I am more and more bubbling up, size is changing, weight is gaining, I look worse in mirror” “I have excessive worries about my baby's health, but I also do not want to get fat”
	Fear of Rejection and Shame Felt	“I fear Body shaming and rejection” “I avoid taking selfies and pictures” “I feel inferior to other women at an event so I prefer not to go” “I feel degraded when people look at me and I feel uncertain about the physical changes occurring to my body” “I feel insecure because my husband doesn’t find me attractive since I have gained weight”
	Concern for Overweight and Body Shape	“I feel Overweight” “I have a strong desire to be thin and no more this chubby.” “I feel I am losing control over my size.”
Lack of recognition of low weight and lack of weight gain	Low weight and Weight gain during pregnancy	“I feel I am ok to manage my routines, I don’t need changes in my eating patterns” “I have gained more than enough weight and I am pretty upset” “I need to be thinner than this, I am upset and this baby bump is way too heavy on my body”
Social Avoidance and Eating to match the norm of eating	Avoiding presence of elders of the family while taking meals	“The elders in the family at my home or in-laws always keep nagging for eating. They want the baby to be healthy but they want me to eat high calorie food, which I don’t want to” “I sneak out of dining area and take food to my room to eat”
	Avoiding eating in gatherings	“I feel uncomfortable and pressurized to eat in social gatherings. This makes me stay at home or isolated sections most of the time. I sneak out of situations. Sometimes when I go out in restaurants I find a corner table or a section where not most people are seated. I prefer not eat on dining table with family just side step. I sneak out of dining area and take food to my room to eat"
	Eating alone to limit food portion	“I prefer to eat alone, what goes in my body is my choice” “When I eat alone there are less chances of my family to policing me”
	Supervised eating by elders being pregnant and later self-induced vomiting	“Eating food is not an issue because I know I can easily induce vomiting when no one is looking” “There is no escape from eating when family members are around. But no matter what amount I am made to eat, I puke afterwards”
	Pretending to Eat suitable amount of food	“I eat eating slowly and consuming more time. Others around are not on my head then to eat more and more.” “I fill up my plate and go in backyard. There I feed some portion of my food to free birds there” “It is easy to hide not eating much but then answering the details of portion and foods taken is difficult. So, I just fill my plate and roam here and there and I take liquids like shakes and drinks”

## Discussion

Pregnancy is considered a maturation crisis, which may encompass phases of weakening of mental and physical defense mechanisms, transformations of self-image, and many conflicts interlinked with femininity. So, many of such pregnancies are psychologically risky and may have somatic problems as well. Women may undergo anxiety, stress, depression, or other psychological problems due to various aspects of pregnancy [31]. Therefore, it can be said that pregnancy is embedded with higher emotional states which may have a negative influence on the fetus.

Pregorexia is a drive in women to control pregnancy weight gain through various methods like extreme dieting and exercise. Many obstetricians do not recognize pregorexia as a psychological disorder therefore, most of the time such women do not get appropriate psychological care to deal with the problem. However, the associated behaviors are real and may have a negative impact on fetal health [24]. Much known in the literature regarding pregorexia is that women excessively care about their weight gain and body image during pregnancy. However, the relevant behaviors that cluster around it are not well-known. Therefore, the present study endeavored to identify the behaviors related to the phenomenon of pregorexia in Pakistan which may highlight what behaviors may be considered as indications for the presence of Pregorexia within Pakistani culture.

The present study found the lived experiences and Behaviors related to Pregorexia which were identified through interviews made with the participants.

### Theme 1: meal avoidance

All participants of the study reported skipping meals in one way or other. Reports included that women indulge in other behaviors to avoid taking their meals like oversleeping or overworking just to keep their minds off the food or hunger. Else they take tiny portions of the meal and take them slowly. Findings are consistent with a study that indicated that 3.5% of women engaged in behaviors like diet restriction that are particularly unhealthy because of the pregnancy [34]. It is typical behavior of some pregnant women to become preoccupied with the idea of controlling weight by taking an extremely low caloric diet and physical exercises. The prevalence rate of pregorexia is 5% among women during pregnancy or post-pregnancy [7].

### Theme 2: diet control

In the case of pregnant women, an impoverished body image is frequently associated with an inadequate or restrictive diet [8–10] which can have serious negative consequences both for the health and well-being of the mother as well as for the fetus [13].

The findings of the present study revealed that women take various measures to control gaining weight. Some women reported to have some knowledge of calorie intake, so they put in efforts to take low-calorie diets. They follow information available on websites and youtube. Their diet plans are not supervised diet plans and it may bring negative consequences equally for mother and fetus. Therefore, there is a need to keep a check and balance for their diet intake. Findings are in line with the literature pregorexia involves self-starvation and/or inducing vomiting during pregnancy (Harasim-Piszczatowska & Krajewska-Kulak). A research confirmed that the intent behind the restriction to food intake is important to be considered in pregnant women to rule out pregorexia, a pregnant woman may restrict to diet at times due to nausea and in many cases, the intent is to minimize or control the gestational weight gain [3].

The eating restraint and concerns regarding weight and body shape in many cases may be healthy or subclinical/ subthreshold (not necessarily disordered). There is much more research needed in this area by utilizing appropriate psychometric and clinical tools. Such cases should be referred to psychiatric clinics for detailed evaluation for a better understanding of the cases and treatment plan. In Pakistan women usually do not exercise, specifically during pregnancy that is why such exercises are alarming and odd behaviors.

### Theme 3: use of products to control weight

The findings indicated that women tend to use various types of products to control their Weight Gain. But the alarming aspect is that many women take these medications irrespective of the fact that they may harm the baby in the fetus. They do it secretively. This might be due to the ease of availability of such medications from drug stores and online shopping websites. Women also take herbal medicines and tea to control their weight. Some women also tend to misuse laxatives and diuretics.

Literature indicates that Diuretics may interfere with the plasma volume expansion after the first trimester which may cause intrauterine growth retardation, low birth weight, and pre-term delivery [28]. The most hazardous behavior of women with pregnancy is the use of laxatives and diuretics which have an adverse impact on not only the mother but also the developing fetus, the adverse consequences may be in form of physical and mental development may have a greater risk for abortion. Perinatal death is more common among the women with low body mass in comparison to their counterparts [15, 32], and women with pregorexia often taken laxatives and diuretics, and other forms of medicines to control their weight gain [26]. The literature only reports about diuretic and laxative use. However, women reported usage of other products too which may not be dangerous but helps them to satisfy the urge to control weight. They also make use of fat-burning products like tea that may help in burning fat. The most popular supplements that may help as fat burners include caffeine, green tea, conjugated linoleic acid, and chromium [17, 39].

### Theme 4: induced vomiting after eating

The findings of the present study indicate that women are often distressed by the idea of getting fat after eating their meal and then they engage in self-induced vomiting, whether there are episodes of binging or no binging but many do purge out food. Results are similar to literature that women who continue to have anxiety regarding their body weight tend to have restricted diet, often use laxatives and diuretics, exercise more frequently, and engage in self-induced vomiting than other groups of pregnant women [30].

The self-induced vomiting is probably linked to the reports of women who indicated the presence of dehydration. A study reported that bulimic pregnant women tend to have more symptoms of dehydration, electrolyte disparity, and more than 5% of body weight is lost. They often require hospitalization [1]. Research reported that medical conditions interlinked with teeth, esophagus, kidneys, and gastrointestinal, cardiovascular, and musculoskeletal systems have a strong association with self-induced vomiting [12]. Present research data reported the presence of medical problems like gastrointestinal problems which may be due to self-induced vomiting.

### Theme 5: physical activities and exercise

The present study showed that women with symptoms of anorexia nervosa tend to have behaviors and activities which may help them stay in shape or reduce weight it was reflective from the quotes of women that they engage in low-impact exercises and activities (walking, light stretching, and swimming), a few women reported indulging in high-impact exercises and activities (cardio) which may be harmful to the health of the baby and may lead to miscarriage. It seems that body image is so much important to them that they disregard the safety of the baby. It may be linked with the history of their abortions. A study in Ireland regarding pregorexia showed that healthy low impact exercises like walking and swimming were consistently prevalent in pre, during, and post-pregnancy stages. However, the high-impact exercises like indoor cycling, cardio, and aerobics were restrained during pregnancy but increased post-delivery of the baby [34].

The results also indicated that exercise is used by such women to manage their worries and thoughts linked to gaining weight. Women with pregorexia have strong concerns for their weight and body size, therefore; So, exercise is a means of coping for them, a good exercise is good to relax and keep fit however an overdo should be avoided by all means not to keep the health of mother and baby at stake. Size-obsessed females can go to extremes to avoid gaining weight during pregnancy, and for compensation, they spend hours at fitness centers, restrict their diet, and risk the health of themselves and their babies [2].

### Theme 6: body dissatisfaction

Research states that most women may experience progress in their behaviors related to eating disorders during pregnancy, but the tension of facing body weight gain, body shape, and body dissatisfaction remains very high, and at times even aggravates [20]. Body dissatisfaction is one of the principal elements of disordered eating and harmful behaviors, therefore, it seems to have a strong relationship with the presence of pregorexia which is absolutely challenging. The present study reported the presence of body dissatisfaction among the women to such an extent that they may keep the life of a baby in the womb at stake. These size and shape-obsessed women can go to extremes to avoid weight gain in pregnancy and to look good in appearance and shape. They have fears to lose body shape and also not being able to get their body shape back. They feel bad looking in the mirror. Their dissatisfaction probably stems out from their fear of rejection, which further influences on their social interaction, self-esteem and sexual relationship with a partner. Body image dissatisfaction correlates strongly with both anorexia nervosa-related behavior and bulimia-related behavior and moderately with binge eating [22].

### Theme 7: lack of recognition of low weight

The findings of the present indicated that women had a lack of recognition of the seriousness of their current low body weight and they also had a lack of understanding that during their first and second trimesters they had not gained appropriate levels of weight. No matter what the doctor or family said they still wanted to get thinner and were not happy for the baby bump. A study reported that losing weight during pregnancy and excessive reassurance to doctors or midwives that low gestational weight or lack of gestational weight gain is nothing to be worried about is found in women with pregorexia [3].

### Theme 8: social avoidance and eating to match the norm of eating

In Pakistan the society is collectivistic and there is a stronghold of elders in the family and children abide by the rules and instructions given by parents or significant elders. Families value affection, solidity, cohesion, and interdependence among their members. Parents are entirely responsible to take care of their children until they get married or sometimes even after marriage if children are not financially stable or need elders like at the time of pregnancy. Similarly, the children take care of their parents, whether living in the same house or apart [25]. When a woman conceives in Pakistan an elder lady who may be a mother, mother-in-law or an elder married sister cares and guides for care for the pregnant lady.

Therefore, the results indicate that the women avoid their elders who ask them to take care and eat well. It is a part of social support and cares in a family that a pregnant lady is taken care of with food (special food is made for her), household chores, preparation of upcoming baby, etc. But these women reported avoiding these elders while eating so that they may easily skip their meals. And if they do not get a chance to avoid them then they eat but later they induce vomiting or they just pretend to eat through the utilization of various tactics like eating slowly, change of place to eat, and feeding their own food to others. It is noted that this supervised eating actually makes them eat else, their health conditions may get deteriorated. The women also indicated that they like to eat alone. Mostly it is seen in bulimia nervosa that people eat alone because they feel embarrassed for the amount of food they eat [37]. However, here it is noted that these women eat alone to limit their food.

The findings suggest that women in Pakistan may be experiencing pregorexia and related interventions are needed to help them. However, there is limited work in this area across the globe and more research is needed. It is significant that the treatment team for pregnant women may be multidisciplinary involving an obstetrician, midwife, psychiatrist, psychologist, dietician, and health trainer. There is a need to set up pregnancy education programs for pregnant women and their husbands, in which education on appropriate nutrition, rules of BMI gain, and side effects of the use of laxatives and other medicines may be a standard of care. Future researchers may screen out such symptoms and tendencies in women pre, during, and post-pregnancy to understand the phenomenon and its occurrence in a better way. In the present study, BMI weight gain was not measured, it is suggested to measure it in order to better assess the severity of these symptoms and prevalence of pregorexia.

A limitation of the study was that the participants were assessed mainly via DSM 5 criteria, their symptoms were cross checked on other eating disorders too. However, as they met the criteria for Anorexia nervosa, so other disorders were not mentioned. Another limitation is that no standardized clinical scale was used for assessment. Future studies may use some standardized tools to understand the phenomenon more accurately. Present study assessed BMI only at one point in time i.e. during the interview. Future studies may report about the changes in the BMI levels before pregnancy, during first trimester, second trimester and third trimester to study the severity levels of the disorder. Due to limitations of hospital settings and interaction time frame questions included in the interview guide were precise and concise (gynae consultants have asked not to take a very lengthy interview). Further, gradations within the society and interactions between these variables have not been discussed and has been added I limitation.

However, future researchers may include inquiries regarding general body image, weight concerns, fat phobia, subjective sense/ objective loss of control over eating behavior, body disparagement, and guilt. The present study entirely focused on lived experiences and behaviors—the cognitive, affective, perceptual, and adaptive aspects of eating pathology have been not adequately covered. Future studies may focus on these aspects to fill the gap in literature.

## Conclusion

There is a need to understand and differentiate pregorexia from other eating behaviors and problems among pregnant women with respect to cultural context. Some pregnant women take gestational weight gain too far and engage in behaviors to minimize their weight gain. They often do not realize that are experiencing a psychological problem and in need of psychological help. Appropriate screening and treatment modalities are needed to be formulated to address the phenomenon. The treatment of this much less known disorder may focus on changing the body image perceptions, body acceptance, education regarding pregnancy and related aspects, psychological care, and relaxation from stress and anxiety.

## Data Availability

The datasets used and/or analyzed during the current study are available from the corresponding author on reasonable request.

## Abbreviations

BMI: Body mass index
BS: Bachelors of Science
Gynae: Gynaecology
